# A retrospective study of the impact of DSM-5 on the diagnosis of eating disorders in Victoria, Australia

**DOI:** 10.1186/s40337-015-0072-0

**Published:** 2015-11-04

**Authors:** Henry Caudle, Christine Pang, Sam Mancuso, David Castle, Richard Newton

**Affiliations:** Austin Hospital, Heidelberg, Australia; University of Melbourne, Parkville, Australia; St Vincent’s Hospital, Melbourne, Australia

**Keywords:** Anorexia nervosa, Bulimia nervosa, EDNOS, OSFED, UFED, Criteria, DSM-5, Eating disorders

## Abstract

**Background:**

This study compares the DSM-IV and DSM-5 diagnostic criteria for eating disorders. DSM-IV resulted in a large number of patients being diagnosed with Eating Disorder Not Otherwise Specified (EDNOS). In DSM-5 the residual category is renamed Other Specified Feeding and Eating Disorders (OSFED) and Unspecified Eating Disorders (UFED) however the diagnostic criteria for the residual category in each of the diagnostic systems remains the same. This study aims to evaluate the changes in percentages of patients in a residual DSM-IV category compared to a residual DSM-5 category by retrospectively applying DSM-5 criteria to the clinical records of a patient population in a clinical setting. It also aims to compare the psychopathology between the EDNOS and OSFED/UFED groups.

**Methods:**

285 participants were recruited from a specialised eating disorder clinic in Australia over a 5-year period from 2009 until 2014. The clinical records of patients with diagnoses of anorexia nervosa (AN), bulimia nervosa (BN) and EDNOS were retrospectively assessed using the DSM-5 criteria. All patients who had attended the clinic and received an eating disorder diagnosis during this period were included in the study. No patients were diagnosed with binge eating disorder during the study period. This is surprising given the prevalence of binge eating disorder in the community. It is possible that individuals with binge eating disorder were not referred to the clinic following the initial referral and assessment due to the lack of binge eating specific interventions available. The referral process may also have been skewed towards AN, BN and EDNOS due to a perception by referring parties that binge eating disorder was a ‘milder’ condition that did not require specialist intervention. Information in the clinical records included structured clinical interviews, and self-rating scales of eating disorder and other psychiatric symptoms and a longitudinal narrative of patient performance and attitude during observed meals.

**Results:**

We observed a 23.5% reduction in the diagnosis of OSFED/UFED with the implementation of DSM-5 compared to EDNOS with DSM-IV. The removal of Criterion D, amenorrhoea, was the leading cause for transition from EDNOS to AN.

**Conclusions:**

DSM-5 has reduced the reliance on EDNOS. However this study was unable to examine the reliability of the new diagnostic criteria or the impact of DSM-5 on binge eating disorder.

## Background

The DSM-IV classification system for eating disorders relied heavily on EDNOS as a residual category for individuals who did not meet the full criteria for AN or BN, with EDNOS accounting for over half of the eating disorder cases in clinical settings [1]. The lack of specific inclusion and exclusion criteria has led to EDNOS being neglected in eating disorder research despite being equally severe [2], having the same distinctive behaviours and psychiatric comorbidities [3], and having the least stability over time compared to other eating disorder diagnoses [4]. DSM-5 attempts to address this problem with the majority of the changes being made to the diagnostic criteria for AN (Table 1).

**Table 1:** Differences between DSM-IV-TR and DSM-5 diagnostic criteria for anorexia nervosa

DSM-IV-TR Diagnostic Criteria	DSM-5 Diagnostic Criteria
A. Refusal to maintain body weight at or above a minimally normal weight for age and height (e.g., weight loss leading to maintenance of body weight less than 85% of that expected; or failure to make expected weight gain during period of growth, leading to body weight less than 85% of that expected).	A. Persistent restriction of energy intake leading to significantly low body weight (in context of what is minimally expected for age, sex, developmental trajectory, and physical health)
B. Intense fear of gaining weight or becoming fat, even though underweight.	B. Either an intense fear of gaining weight or of becoming fat, or persistent behaviour that interferes with weight gain (even though significantly low weight).
C. Disturbance in the way in which one’s body weight or shape is experienced, undue influence of body weight or shape on self-evaluation, or denial of the seriousness of the current low body weight.	C. Disturbance in the way one’s body weight or shape is experienced, undue influence of body shape and weight on self-evaluation, or persistent lack of recognition of the seriousness of the current low body weight.
D. In postmenarcheal females, amenorrhea, i.e., the absence of at least three consecutive menstrual cycles. (A woman is considered to have amenorrhea if her periods occur only following hormone, e.g., oestrogen administration.)	

*DSM-IV-TR* Diagnostic and Statistical Manual of Mental Disorders, fourth edition, Text Revision, *DSM-5* Diagnostic and Statistical Manual of Mental Disorder, fifth edition

DSM-5 no longer stipulates a specific BMI for criterion A. Criterion B now recognises ‘persistent behaviour that interferes with weight gain’ and criterion C recognises ‘persistent lack of recognition of the seriousness of the current low body weight’. Criterion D (amenorrhoea) has been removed.

Several studies have shown that despite lowering the threshold for diagnosis, DSM-5 is better able to differentiate groups of eating disorders than DSM-IV [3, 4]. However few studies have investigated the impact of DSM-5 on the frequency of unspecified eating disorders. The first aim of this study is to use DSM-IV and DSM-5 to diagnose eating disorders and evaluate the change in proportion of patients in each group. The second aim is to compare psychopathology between the EDNOS and OSFED groups.

## Methods

Data came from 285 consecutive patients over the age of 17 who attended an outpatient clinic for eating disorders in Melbourne, Australia, from 2009 to 2014. All patients who were diagnosed with a DSM-IV eating disorder were included in the study. The majority of participants were Australian born females from an Anglo-Saxon background, which could limit the application of these findings to other populations.

Clinicians trained in using a structured documentation suite and the Mini International Neuropsychiatric Interview (MINI) Version ICD-10 interviewed the participants at the time of the initial assessment. Behaviour of participants around meal times and cooperation with the program was documented in the clinical file. A single researcher reviewed this data and the clinical notes retrospectively.

After reviewing the files, DSM-5 and DSM-IV criteria were applied to all participants retrospectively. In this study we considered patients with a BMI <18.5 kg/m^2^ to fulfil DSM-5 criterion A, as well as those with a BMI over 18.5 kg/m^2^ who could still be considered to be at a significantly low weight due to persistent restriction of energy intake. It was decided to include these individuals who had rapid weight loss or high premorbid weight in the AN group due to the similarities with the rest of the AN group in every other respect. The authors note that atypical AN would be an alternate way of describing these individuals, however for the purpose of this study only the differentiation from OSFED/UFED is relevant. The documentation of mealtime behaviours such as persistent behaviour that interferes with weight gain and attitude to the seriousness of a low bodyweight enabled this information to be retrospectively applied to DSM-5 criteria.

Ethics approval was obtained from the St Vincent’s Human Resource Ethics Committee. The authors declare that they have no competing interests.

STATA 12.1 (StataCorp, College Station, Tx) was used to analyse the data.

## Results

### Patient demographics

285 patients were included in the study, 269 (94.3%) females and 16 (5.7%) males. The age of the patients ranged from 17 to 78 years with a mean age of 26. The average BMI at assessment was 17.9 kg/m^2^. Patients who did not attract an eating disorder diagnosis were not included in the study. The BMI ranges are presented in Table 2

**Table 2:** The frequency (n) and percentage (%) of BMI ranges in all participants that attended BETRS for assessment

BMI Range (kg/m^2)^	n (%)
Underweight (<18.50 kg/m^2^)	
*Severe thinness (<16 kg/m* ^*2*^ *)*	88 (30.9)
*Moderate thinness (16–16.99 kg/m* ^*2*^ *)*	34 (11.9)
*Mild thinness (17.00-18.49 kg/m* ^*2*^ *)*	57 (20.0)
Normal (18.50-24.99 kg/m^2^)	93 (32.6)
Overweight (25–29.99 kg/m^2^)	13 (4.6)
Obese (>30 kg/m^2^)	0 (0)

*BMI* Body Mass Index, *BETRS* Body Image & Eating Disorders Treatment and Recovery Service, *n* number of patients

### The impact of DSM-5 on EDNOS

Table 3 describes the number of diagnoses of AN, BN, EDNOS and OSFED/UFED after application of the DSM-IV and DSM-5 criteria. DSM-5 led to a 25.2% increase in AN, a 0.7% increase in BN, and a 23.5% decrease in the diagnosis of EDNOS (OSFED).

**Table 3:** Frequency (n) and percentage (%) changes in the diagnoses of AN, BN, EDNOS and OSFED/UFED with the application of DSM-IV and DSM-5 criteria

	DSM-IV		DSM-5		% Change
	n	%	n	%	
AN	130	45.6	195	68.4	25.2 (*p* < 0.001)
BN	56	19.6	58	20.4	0.7 (*p* = 0.15)
EDNOS/OSFED/UFED	99	34.7	38.7	11.2	23.5 (*p* < 0.001)

*DSM-IV TR* Diagnostic and Statistical Manual of Mental Disorders, fourth edition, Text Revision, *DSM-5* Diagnostic and Statistical Manual of Mental Disorder, fifth edition, *AN* Anorexia Nervosa, *BN* bulimia nervosa, *EDNOS* eating disorder not otherwise specified, *OSFED* other specified eating disorder, *UFED* unspecified eating disorder

### Transition from EDNOS (DSM-IV) to AN (DSM-5)

65 patients transitioned from EDNOS (DSM-IV) to AN (DSM-5). 49 participants who did not meet the criterion D for amenorrhoea in DSM-IV were subsequently diagnosed with AN under DSM-5. 19 participants that had not previously met criterion A in DSM-IV did so with DSM-5. 21 participants met the definition of criterion B under DSM-5 who had not under DSM-IV, and 12 participants met criterion C who had not under DSM-IV. The change in frequency in which criterion C was assessed as being fulfilled could have been due to a change in which this criterion is worded in DSM-5, possibly allowing for a more objective interpretation of ‘lack of recognition’ compared to ‘denial of’ the seriousness of the low body weight between the two classification systems. The authors acknowledge that there is considerable overlap between these groups. These results are represented in Table 4.

**Table 4:** Number (n) and percentage (%) of patients where a criterion was not fulfilled under DSM-IV-TR who transitioned to a diagnosis of AN after applying DSM-5

DSM-IV-TR AN	n	%
Criterion A	19	29.2
Criterion B	21	32.3
Criterion C	12	18.5
Criterion D^a^	49	75.4

*DSM-IV TR* Diagnostic and Statistical Manual of Mental Disorders, fourth edition, Text Revision, *DSM-5* Diagnostic and Statistical Manual of Mental Disorder, fifth edition, *AN* anorexia nervosa

^a^Criterion D not present in DSM-5

### Differences in EDNOS and OSFED group characteristics

99 participants were diagnosed with EDNOS using the DSM-IV classification system. The average age was 25.4 years and the average BMI was 17.9 kg/m^2^. Using DSM-5, 32 participants were diagnosed with OSFED/UFED with an average age of 27.3 years and an average BMI of 19.3 kg/m^2^.

### Differences in EDNOS and OSFED/UFED psychiatric comorbidities

The rates of psychiatric comorbidities are presented in Table 5. Most psychiatric comorbities were represented in relatively similar numbers, however obsessive-compulsive disorder and dysthymia was substantially more common in the EDNOS group than the OSFED/UFED group.

**Table 5:** MINI Version ICD-10 results reflecting psychiatric comorbidities in EDNOS and OSFED/UFED groups

	DSM-IV EDNOS	DSM-5 OSFED/UFED
Diagnosis	n (%)	n (%)
Depressive Episode	43 (53.1)	14 (53.8)
Recurrent Depressive Disorder	28 (34.6)	9 (34.6)
Dysthymia	19 (23.5)	1 (3.8)
Manic Episode (Lifetime)	5 (6.2)	1 (3.8)
Agoraphobia	14 (17.3)	5 (19.2)
Panic Disorder	30 (37.0)	8 (30.8)
Agoraphobia with Panic Disorder	2 (2.5)	2 (7.7)
Social Phobia	18 (22.2)	4 (15.4)
Obsessive-Compulsive Disorder	15 (18.5)	2 (7.7)
Generalized Anxiety Disorder	21 (25.9)	7 (26.9)
Post-traumatic Stress Disorder	16 (19.8)	4 (15.4)
Alcohol Dependence	10 (12.3)	3 (11.5)
Harmful Use of Alcohol	1 (1.2)	1 (3.8)
Drug(s) Dependence	11 (13.6)	3 (11.5)
Harmful Use of Alcohol	1 (1.2)	1 (3.8)
Single Psychotic Episode (Lifetime)	6 (7.4)	1 (3.8)
Recurrent Psychotic Episode (Lifetime)	7 (8.6)	3 (11.5)

*EDNOS* eating disorder not otherwise specified, *OSFED* other specified eating disorder, *UFED* unspecified eating disorder, *DSM-IV TR* Diagnostic and Statistical Manual of Mental Disorders, fourth edition, Text Revision, *DSM-5* Diagnostic and Statistical Manual of Mental Disorder, fifth edition

### Differences in EDNOS and OSFED/UFED baseline self-report questionnaires

A set of self-report measures from the participants had been collected from the participants during their first assessment.

The results were tabulated and are shown in Table 6. No statistical significance was found between any of the self-report measures (*p* < 0.05).

**Table 6:** Frequency (n), mean *(M)*, standard deviation (SD) and P value, derived from t-tests, comparing the baseline measures in EDNOS and OSFED/UFED

	DSM-IV EDNOS			DSM-5 OSFED/UFED			*P* value
	n	*M*	SD	n	*M*	SD	
EDE-Q	51	4.07	0.16	13	3.24	0.59	0.19
DASS-21							
*Depression*	87	24.46	1.30	31	21.94	2.42	0.41
*Anxiety*	87	20.32	1.19	31	18.65	2.09	0.65
*Stress*	87	26.76	1.23	31	25.10	2.47	0.50
Q-LES-Q-	76	39.92	2.05	28	45.55	3.35	0.14
URICA-32	76	9.13	0.23	28	9.56	0.43	0.34

EDNOS, eating disorder not otherwise specified; OSFED, other specified eating disorder; UFED, unspecified eating disorder; EDE-Q, Eating Disorder Examination Questionnaire; DASS-21, Depression, Anxiety and Stress Scale – 21; Q-LES-Q, Quality of Life Enjoyment and Satisfaction Questionnaire; University of Rhode Island Change Assessment Scale - 32

## Discussion

This study investigated the changes in rates of AN, BN and EDNOS diagnoses after applying the DSM-5 eating disorder criteria to a clinical sample. Consistent with previous research, the DSM-5 criteria resulted in a significant increase in anorexia diagnosis and a reduction in the diagnosis of residual clinically significant eating disorders that did not fit a specific diagnostic category [5]. Our study found an increase of 25.2% in the diagnosis of AN, a reduction of 23.5% of EDNOS (renamed OSFED/UFED in DSM-5) and a minimal change in BN (0.8% increase).

Our results demonstrate that DSM-5 has reduced the reliance on EDNOS. Studies have found that despite lowering the threshold for diagnosis of anorexia nervosa, DSM-5 has been able to characterize eating disorders more precisely than DSM-IV by reducing the reliance on a residual category [5]. Our findings are comparable to other research studies that have utilized clinical samples from America, Portugal, Sweden and Japan. These studies have demonstrated a reduction in EDNOS rates ranging from17.0% to 22.1% [5–8].

The removal of criterion D (amenorrhoea) resulted in the largest number of transitions from EDNOS to AN, which is consistent with several other studies [6, 9]. However studies have also suggested that inclusion of binge eating disorder into DSM-5 has also contributed to a significant reduction in EDNOS diagnosis [6, 10].

Our study was unique in that we were able to retrospectively analyse all the criterion changes in AN to demonstrate the impact of DSM-5 on the rates of AN, BN and EDNOS. However the retrospective nature of the study creates limitations in standardisation of the information collected during the initial assessments and vulnerability to problems with inter-rater reliability. There is also a potential for investigator bias, which could exaggerate the number of participants fulfilling criterion C under DSM-5 but not under DSM-IV. Other studies have reported difficulties in interpreting changes in criterion B - persistent behaviour that interferes with weight gain – due to limitations of the Eating Disorder Examination Questionnaire (EDE-Q) in capturing these changes [5, 7–9]. Our study was able to overcome this problem by utilizing data collected through face-to-face clinical interviews and the practice of documenting mealtime behaviours after every meal.

We avoided setting a specific BMI cut-off for the change to Criterion A. The DSM-5 criterion A for anorexia requires patients to have a ‘significantly low body weight’. This change was based on evidence that there is considerable variation in the bodyweight that is associated with the harmful consequences of starvation. However, this could potentially lead to inconsistency among research.

Several studies have interpreted this as a threshold by setting a BMI <17.5 kg/m^2^ as the cut-off in their studies [5–7]. Other studies have kept the BMI <18.5 kg/m^2^ as the cut-off [6, 9]. Our study included any participant with a BMI < 18.5 kg/m^2^ but also considered participants who had a BMI >18.5 kg/m^2^ who could be considered to be at a significantly low weight and who actively restricted their energy intake.

The changes to Criterion B have been difficult to identify in previous studies. Our study relied on documented behaviours observed longitudinally during the outpatient program, and was able to establish criterion B when the patient had not articulated a fear of weight gain. Patients with a low BMI who failed to fulfil the other DSM-IV criteria may have been reclassified from EDNOS to AN under DSM-5 given the broader classification system. This could account for the average BMI of OSFED patients being higher than the EDNOS group.

We compared the DSM-IV EDNOS group of participants with the DSM-5 OSFED/UFED group on basic clinical variables, comorbid psychiatric disorders and baseline self-report measures. The average age and the average BMI was higher in the DSM-5 OSFED/UFED group. We found a minimal change in percentages of participants diagnosed with a comorbid psychiatric disorder using the MINI Version ICD-10 diagnostic tool. As shown in Table 5, the only exceptions were a significant reduction in the incidence of dysthymia and Obsessive-Compulsive Disorder the DSM-5 OSFED/UFED group. It is possible that certain co-morbidities track with the AN phenotype and hence moved into the AN group under DSM-5 resulting in a reduction of these co-morbidities in the OSFED/UFED group. There were no significant differences between baseline self-report measures (Table 6). No participant transitioned from a DSM-IV EDNOS diagnosis to a DSM-5 binge eating disorder diagnosis. This could be a consequence of the population who access this particular service or limitations in the understanding of binge eating as an eating disorder diagnosis by referring clinicians.

The limitations of this study are that it is a retrospective analysis of clinical notes which could not control for variability in the quality of documentation and inter-rater reliability of the initial assessments. The results may not be generalizable to all populations or cultural groups due the demographics of participants accessing this particular service.

## Conclusions

Our study was the first to incorporate all changes in the DSM-5 diagnostic criteria of AN in clinical setting. We demonstrated a reduction in reliance on the residual eating disorder categories by 23.5% and did not observe significant difference in psychopathology between the EDNOS group and OSFED/UFED groups.
